# Diphenyl­methyl isothio­cyanate

**DOI:** 10.1107/S1600536812000888

**Published:** 2012-01-14

**Authors:** Pei-Hua Zhao, Jun-Jie Liu, Mei Zhang, Gui-Zhe Zhao, Ya-Qing Liu

**Affiliations:** a Research Center for Engineering Technology of Polymeric Composites of Shanxi Province, School of Materials Science and Engineering, North University of China, Taiyuan 030051, People’s Republic of China

## Abstract

The asymmetric unit of the title compound, C_14_H_11_NS, contains two mol­ecules in which the dihedral angles between the phenyl rings are 77.23 (7) and 86.30 (7)°. No aromatic π–π stacking inter­actions are observed.

## Related literature

For the synthetic applications of isothio­cyanates, see: Fernandez *et al.* (1995 ▶); Mukerjee & Ashare (1991 ▶); Stephensen & Zaragosa (1997 ▶).
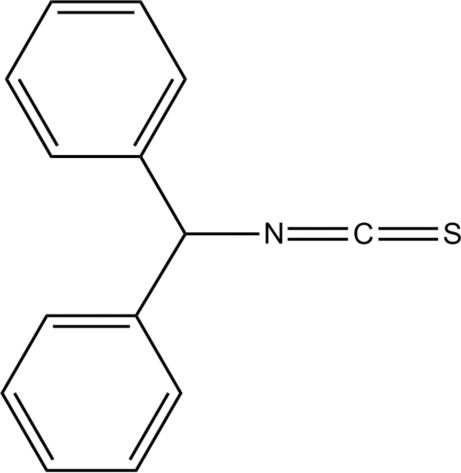


## Experimental

### 

#### Crystal data

C_14_H_11_NS
*M* *_r_* = 225.30
Triclinic, 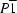

*a* = 9.635 (5) Å
*b* = 10.222 (6) Å
*c* = 11.974 (7) Å
α = 98.491 (13)°
β = 95.296 (15)°
γ = 93.573 (6)°
*V* = 1157.9 (11) Å^3^
*Z* = 4
Mo *K*α radiation
μ = 0.25 mm^−1^
*T* = 113 K
0.24 × 0.20 × 0.18 mm

#### Data collection

Rigaku Saturn724 CCD diffractometer
Absorption correction: multi-scan (*CrystalClear*; Rigaku/MSC, 2005 ▶) *T*_min_ = 0.943, *T*_max_ = 0.957
12035 measured reflections
5430 independent reflections
3169 reflections with *I* > 2σ(*I*)
*R*_int_ = 0.042

#### Refinement

*R*[*F* ^2^ > 2σ(*F* ^2^)] = 0.034
*wR*(*F* ^2^) = 0.079
*S* = 0.89
5430 reflections
289 parameters
2 restraints
H-atom parameters constrained
Δρ_max_ = 0.20 e Å^−3^
Δρ_min_ = −0.26 e Å^−3^

### 

Data collection: *CrystalClear* (Rigaku/MSC, 2005 ▶); cell refinement: *CrystalClear*; data reduction: *CrystalClear*; program(s) used to solve structure: *SHELXS97* (Sheldrick, 2008 ▶); program(s) used to refine structure: *SHELXL97* (Sheldrick, 2008 ▶); molecular graphics: *SHELXTL* (Sheldrick, 2008 ▶); software used to prepare material for publication: *SHELXL97*.

## Supplementary Material

Crystal structure: contains datablock(s) global, I. DOI: 10.1107/S1600536812000888/hb6596sup1.cif

Structure factors: contains datablock(s) I. DOI: 10.1107/S1600536812000888/hb6596Isup2.hkl

Supplementary material file. DOI: 10.1107/S1600536812000888/hb6596Isup3.cml

Additional supplementary materials:  crystallographic information; 3D view; checkCIF report

## Figures and Tables

**Fig. 1.**
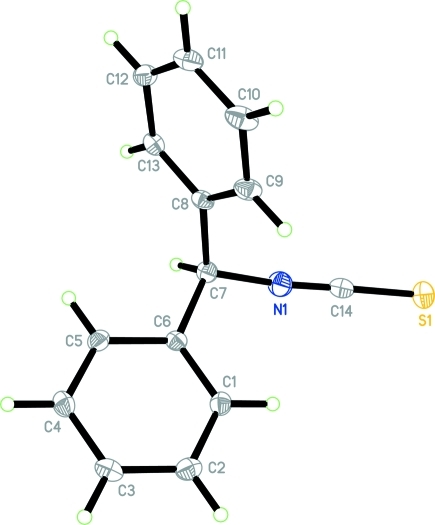
The molecular structure of (I). Displacement ellipsoids are drawn at the 30% probability level and H atoms are shown as small spheres of arbitrary radii.

**Fig. 2.**
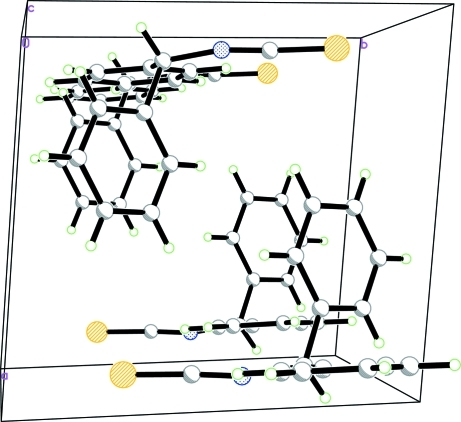
The crystal packing for (I).

## supplementary crystallographic information

### Experimental

Diphenylamine (44.0 mmol) was dissolved in absolute ethanol (50.0 ml). Carbon disulfide (440.0 mmol) and triethylamine (44.0 mmol) were added while stirring. The reaction mixture was stirred for 0.5 h at room temperature and then cooled on an ice bath. Di-*tert* butyl dicarbonate (43.6 mmol) dissolved in absolute ethanol (10.0 ml), was added followed by the immediate addition of a catalytic amount of 1,4-diazabicyclo-[2.2.2]octane (0.88 mmol) in absolute ethanol (10.0 ml). The reaction mixture was kept in the ice bath for 5 min, and was then allowed to room temperature. After the reaction was completed, the solvents were evporated thoroughly *in vacuo*. The residue obtained was taken up in ether and filtered off, and the filtrate was evaporated in vacuo to afford the crude. The crude was separated through column chromatography on silica gel eluting with petroleum ether- dichloromethane (30:1 *v*/*v*) to give the white product. Colourless prisms of the title compound were obtained by slow evaporation of the dichloromethane/n-hexane solutions at room temperature. ^1^H-NMR(400 MHz, CDCl_3_, TMS): 6.02 (s, 1H, CH), 7.33–7.42 (m, 10H, Ph—H) p.p.m.. ^13^C-NMR(100 MHz,CDCl_3_, TMS): 64.6 (CH), 126.7, 128.4, 129.0, 139.3 (Ph—CH and Ph—C) p.p.m..

### Refinement

All the H atoms were positioned geometrically (C—H = 0.95 Å) and refined as riding with *U*_iso_(H) = 1.2*U*_eq_(C).

### Crystal data

**Table:**

C_14_H_11_NS	*Z* = 4
*M**_r_* = 225.30	*F*(000) = 472
Triclinic, *P*1	*D*_x_ = 1.292 Mg m^−^^3^
*a* = 9.635 (5) Å	Mo *K*α radiation, λ = 0.71073 Å
*b* = 10.222 (6) Å	Cell parameters from 3900 reflections
*c* = 11.974 (7) Å	θ = 1.7–28.0°
α = 98.491 (13)°	µ = 0.25 mm^−^^1^
β = 95.296 (15)°	*T* = 113 K
γ = 93.573 (6)°	Prism, colorless
*V* = 1157.9 (11) Å^3^	0.24 × 0.20 × 0.18 mm

### Data collection

**Table:**

Rigaku Saturn724 CCD diffractometer	5430 independent reflections
Radiation source: rotating anode	3169 reflections with *I* > 2σ(*I*)
multilayer	*R*_int_ = 0.042
Detector resolution: 14.22 pixels mm^-1^	θ_max_ = 27.9°, θ_min_ = 1.7°
ω and φ scans	*h* = −12→12
Absorption correction: multi-scan (*CrystalClear*; Rigaku/MSC, 2005)	*k* = −13→13
*T*_min_ = 0.943, *T*_max_ = 0.957	*l* = −15→12
12035 measured reflections	

### Refinement

**Table:**

Refinement on *F*^2^	Primary atom site location: structure-invariant direct methods
Least-squares matrix: full	Secondary atom site location: difference Fourier map
*R*[*F*^2^ > 2σ(*F*^2^)] = 0.034	Hydrogen site location: inferred from neighbouring sites
*wR*(*F*^2^) = 0.079	H-atom parameters constrained
*S* = 0.89	*w* = 1/[σ^2^(*F*_o_^2^) + (0.0253*P*)^2^] where *P* = (*F*_o_^2^ + 2*F*_c_^2^)/3
5430 reflections	(Δ/σ)_max_ = 0.003
289 parameters	Δρ_max_ = 0.20 e Å^−^^3^
2 restraints	Δρ_min_ = −0.26 e Å^−^^3^

### Special details

**Table:**

Geometry. All e.s.d.'s (except the e.s.d. in the dihedral angle between two l.s. planes) are estimated using the full covariance matrix. The cell e.s.d.'s are taken into account individually in the estimation of e.s.d.'s in distances, angles and torsion angles; correlations between e.s.d.'s in cell parameters are only used when they are defined by crystal symmetry. An approximate (isotropic) treatment of cell e.s.d.'s is used for estimating e.s.d.'s involving l.s. planes.
Refinement. Refinement of *F*^2^ against ALL reflections. The weighted *R*-factor *wR* and goodness of fit *S* are based on *F*^2^, conventional *R*-factors *R* are based on *F*, with *F* set to zero for negative *F*^2^. The threshold expression of *F*^2^ > σ(*F*^2^) is used only for calculating *R*-factors(gt) *etc*. and is not relevant to the choice of reflections for refinement. *R*-factors based on *F*^2^ are statistically about twice as large as those based on *F*, and *R*- factors based on ALL data will be even larger.

### Fractional atomic coordinates and isotropic or equivalent isotropic displacement parameters (Å^2^)

**Table:**

	*x*	*y*	*z*	*U*_iso_*/*U*_eq_	
S1	0.10665 (4)	0.73024 (4)	0.96006 (4)	0.03299 (12)	
S2	0.11876 (4)	1.01973 (4)	0.18909 (4)	0.03186 (12)	
N1	0.10403 (14)	0.46640 (15)	0.86731 (12)	0.0375 (4)	
N2	0.27245 (15)	0.85453 (13)	0.30434 (11)	0.0353 (4)	
C1	0.14265 (15)	0.41926 (16)	0.63396 (13)	0.0266 (4)	
H1	0.1367	0.5072	0.6711	0.032*	
C2	0.15692 (15)	0.39629 (17)	0.51813 (14)	0.0298 (4)	
H2	0.1605	0.4686	0.4766	0.036*	
C3	0.16580 (15)	0.26911 (17)	0.46354 (13)	0.0286 (4)	
H3	0.1770	0.2538	0.3848	0.034*	
C4	0.15828 (15)	0.16335 (16)	0.52411 (13)	0.0292 (4)	
H4	0.1623	0.0754	0.4863	0.035*	
C5	0.14493 (15)	0.18588 (15)	0.63921 (13)	0.0267 (4)	
H5	0.1411	0.1133	0.6803	0.032*	
C6	0.13704 (14)	0.31449 (15)	0.69539 (12)	0.0216 (3)	
C7	0.12841 (16)	0.33178 (15)	0.82319 (12)	0.0254 (4)	
H7	0.0469	0.2731	0.8372	0.030*	
C8	0.25897 (15)	0.28997 (15)	0.88646 (12)	0.0233 (3)	
C9	0.38754 (16)	0.36001 (17)	0.88849 (13)	0.0309 (4)	
H9	0.3937	0.4374	0.8534	0.037*	
C10	0.50688 (16)	0.31767 (18)	0.94139 (13)	0.0352 (4)	
H10	0.5947	0.3656	0.9421	0.042*	
C11	0.49818 (16)	0.20569 (17)	0.99316 (13)	0.0324 (4)	
H11	0.5800	0.1768	1.0296	0.039*	
C12	0.37035 (17)	0.13574 (16)	0.99191 (13)	0.0309 (4)	
H12	0.3645	0.0585	1.0272	0.037*	
C13	0.25053 (16)	0.17819 (15)	0.93923 (12)	0.0259 (4)	
H13	0.1627	0.1306	0.9393	0.031*	
C14	0.10830 (15)	0.57794 (17)	0.90672 (13)	0.0262 (4)	
C15	0.25922 (15)	0.57519 (16)	0.25156 (13)	0.0269 (4)	
H15	0.1838	0.6219	0.2249	0.032*	
C16	0.26212 (16)	0.44037 (16)	0.21331 (13)	0.0305 (4)	
H16	0.1888	0.3949	0.1607	0.037*	
C17	0.37235 (16)	0.37218 (16)	0.25209 (13)	0.0284 (4)	
H17	0.3748	0.2801	0.2256	0.034*	
C18	0.47868 (16)	0.43822 (15)	0.32921 (13)	0.0260 (4)	
H18	0.5539	0.3914	0.3560	0.031*	
C19	0.47557 (15)	0.57276 (15)	0.36751 (12)	0.0241 (3)	
H19	0.5486	0.6178	0.4206	0.029*	
C20	0.36602 (15)	0.64199 (14)	0.32856 (12)	0.0208 (3)	
C21	0.37093 (15)	0.79000 (14)	0.37246 (12)	0.0242 (4)	
H21	0.4669	0.8293	0.3656	0.029*	
C22	0.34411 (15)	0.81973 (14)	0.49715 (13)	0.0215 (3)	
C23	0.45057 (16)	0.87830 (14)	0.57871 (13)	0.0249 (4)	
H23	0.5404	0.9000	0.5567	0.030*	
C24	0.42671 (16)	0.90538 (15)	0.69248 (13)	0.0280 (4)	
H24	0.4997	0.9462	0.7478	0.034*	
C25	0.29602 (16)	0.87249 (15)	0.72478 (13)	0.0277 (4)	
H25	0.2791	0.8911	0.8022	0.033*	
C26	0.19007 (16)	0.81236 (15)	0.64359 (13)	0.0279 (4)	
H26	0.1009	0.7890	0.6659	0.034*	
C27	0.21326 (15)	0.78614 (15)	0.53047 (13)	0.0249 (4)	
H27	0.1401	0.7452	0.4754	0.030*	
C28	0.20928 (15)	0.92584 (15)	0.25600 (12)	0.0226 (3)	

### Atomic displacement parameters (Å^2^)

**Table:**

	*U*^11^	*U*^22^	*U*^33^	*U*^12^	*U*^13^	*U*^23^
S1	0.0286 (2)	0.0308 (3)	0.0386 (3)	0.00210 (18)	0.00829 (19)	−0.0008 (2)
S2	0.0346 (2)	0.0281 (2)	0.0330 (3)	0.00572 (18)	−0.00353 (19)	0.0080 (2)
N1	0.0422 (9)	0.0355 (9)	0.0344 (9)	0.0162 (7)	0.0031 (7)	−0.0012 (7)
N2	0.0481 (9)	0.0309 (8)	0.0290 (8)	0.0109 (7)	0.0020 (7)	0.0090 (7)
C1	0.0235 (8)	0.0244 (9)	0.0313 (10)	0.0030 (7)	0.0002 (7)	0.0029 (8)
C2	0.0246 (9)	0.0332 (10)	0.0326 (10)	−0.0012 (7)	−0.0005 (7)	0.0120 (8)
C3	0.0204 (8)	0.0427 (11)	0.0220 (9)	−0.0013 (7)	0.0023 (7)	0.0047 (8)
C4	0.0292 (9)	0.0283 (9)	0.0285 (10)	0.0042 (7)	0.0036 (7)	−0.0023 (8)
C5	0.0292 (9)	0.0250 (9)	0.0271 (9)	0.0066 (7)	0.0025 (7)	0.0064 (8)
C6	0.0167 (8)	0.0251 (9)	0.0228 (9)	0.0050 (6)	0.0004 (6)	0.0031 (7)
C7	0.0245 (8)	0.0270 (9)	0.0247 (9)	0.0053 (7)	0.0034 (7)	0.0020 (7)
C8	0.0232 (8)	0.0298 (9)	0.0163 (8)	0.0047 (7)	0.0023 (6)	0.0006 (7)
C9	0.0312 (9)	0.0410 (11)	0.0218 (9)	−0.0020 (8)	0.0031 (7)	0.0106 (8)
C10	0.0230 (9)	0.0561 (12)	0.0264 (10)	−0.0036 (8)	0.0018 (7)	0.0096 (9)
C11	0.0275 (9)	0.0463 (12)	0.0230 (9)	0.0102 (8)	−0.0007 (7)	0.0032 (8)
C12	0.0380 (10)	0.0300 (10)	0.0250 (9)	0.0085 (8)	0.0008 (8)	0.0045 (8)
C13	0.0253 (9)	0.0283 (9)	0.0225 (9)	0.0002 (7)	0.0027 (7)	−0.0003 (7)
C14	0.0200 (8)	0.0382 (11)	0.0218 (9)	0.0081 (7)	0.0047 (6)	0.0052 (8)
C15	0.0214 (8)	0.0314 (10)	0.0277 (9)	0.0040 (7)	0.0001 (7)	0.0048 (8)
C16	0.0281 (9)	0.0335 (10)	0.0259 (9)	−0.0053 (7)	−0.0013 (7)	−0.0030 (8)
C17	0.0340 (10)	0.0224 (9)	0.0289 (10)	0.0028 (7)	0.0084 (8)	0.0006 (8)
C18	0.0254 (9)	0.0264 (9)	0.0277 (9)	0.0076 (7)	0.0042 (7)	0.0057 (8)
C19	0.0210 (8)	0.0278 (9)	0.0225 (9)	0.0008 (6)	0.0004 (6)	0.0023 (7)
C20	0.0204 (8)	0.0226 (8)	0.0201 (8)	0.0015 (6)	0.0045 (6)	0.0043 (7)
C21	0.0235 (8)	0.0235 (9)	0.0271 (9)	0.0035 (7)	0.0026 (7)	0.0084 (7)
C22	0.0252 (8)	0.0152 (8)	0.0253 (9)	0.0055 (6)	0.0020 (7)	0.0051 (7)
C23	0.0231 (8)	0.0196 (8)	0.0324 (10)	−0.0002 (6)	0.0011 (7)	0.0071 (7)
C24	0.0311 (9)	0.0229 (9)	0.0278 (10)	−0.0020 (7)	−0.0067 (7)	0.0044 (7)
C25	0.0352 (10)	0.0242 (9)	0.0243 (9)	0.0071 (7)	0.0035 (7)	0.0032 (7)
C26	0.0258 (9)	0.0276 (9)	0.0309 (10)	0.0054 (7)	0.0046 (7)	0.0039 (8)
C27	0.0216 (8)	0.0256 (9)	0.0261 (9)	0.0026 (7)	−0.0016 (7)	0.0013 (7)
C28	0.0255 (8)	0.0212 (8)	0.0203 (9)	−0.0005 (6)	0.0029 (6)	0.0015 (7)

### Geometric parameters (Å, °)

**Table:**

S1—C14	1.5947 (19)	C12—C13	1.388 (2)
S2—C28	1.5893 (16)	C12—H12	0.9500
N1—C14	1.164 (2)	C13—H13	0.9500
N1—C7	1.441 (2)	C15—C20	1.388 (2)
N2—C28	1.1626 (18)	C15—C16	1.389 (2)
N2—C21	1.4473 (19)	C15—H15	0.9500
C1—C6	1.387 (2)	C16—C17	1.388 (2)
C1—C2	1.393 (2)	C16—H16	0.9500
C1—H1	0.9500	C17—C18	1.383 (2)
C2—C3	1.378 (2)	C17—H17	0.9500
C2—H2	0.9500	C18—C19	1.387 (2)
C3—C4	1.390 (2)	C18—H18	0.9500
C3—H3	0.9500	C19—C20	1.390 (2)
C4—C5	1.383 (2)	C19—H19	0.9500
C4—H4	0.9500	C20—C21	1.523 (2)
C5—C6	1.395 (2)	C21—C22	1.528 (2)
C5—H5	0.9500	C21—H21	1.0000
C6—C7	1.525 (2)	C22—C23	1.389 (2)
C7—C8	1.525 (2)	C22—C27	1.3949 (19)
C7—H7	1.0000	C23—C24	1.393 (2)
C8—C13	1.387 (2)	C23—H23	0.9500
C8—C9	1.388 (2)	C24—C25	1.387 (2)
C9—C10	1.385 (2)	C24—H24	0.9500
C9—H9	0.9500	C25—C26	1.388 (2)
C10—C11	1.381 (2)	C25—H25	0.9500
C10—H10	0.9500	C26—C27	1.383 (2)
C11—C12	1.383 (2)	C26—H26	0.9500
C11—H11	0.9500	C27—H27	0.9500
			
C14—N1—C7	168.64 (16)	N1—C14—S1	177.42 (15)
C28—N2—C21	168.05 (17)	C20—C15—C16	120.28 (14)
C6—C1—C2	120.40 (15)	C20—C15—H15	119.9
C6—C1—H1	119.8	C16—C15—H15	119.9
C2—C1—H1	119.8	C17—C16—C15	119.89 (15)
C3—C2—C1	120.23 (15)	C17—C16—H16	120.1
C3—C2—H2	119.9	C15—C16—H16	120.1
C1—C2—H2	119.9	C18—C17—C16	120.02 (15)
C2—C3—C4	119.74 (15)	C18—C17—H17	120.0
C2—C3—H3	120.1	C16—C17—H17	120.0
C4—C3—H3	120.1	C17—C18—C19	120.05 (14)
C5—C4—C3	120.16 (15)	C17—C18—H18	120.0
C5—C4—H4	119.9	C19—C18—H18	120.0
C3—C4—H4	119.9	C18—C19—C20	120.31 (15)
C4—C5—C6	120.48 (15)	C18—C19—H19	119.8
C4—C5—H5	119.8	C20—C19—H19	119.8
C6—C5—H5	119.8	C15—C20—C19	119.45 (15)
C1—C6—C5	118.97 (14)	C15—C20—C21	123.10 (14)
C1—C6—C7	123.65 (14)	C19—C20—C21	117.44 (14)
C5—C6—C7	117.33 (13)	N2—C21—C20	111.07 (13)
N1—C7—C6	111.17 (13)	N2—C21—C22	109.58 (12)
N1—C7—C8	110.31 (13)	C20—C21—C22	112.88 (12)
C6—C7—C8	111.92 (12)	N2—C21—H21	107.7
N1—C7—H7	107.8	C20—C21—H21	107.7
C6—C7—H7	107.8	C22—C21—H21	107.7
C8—C7—H7	107.8	C23—C22—C27	119.35 (14)
C13—C8—C9	119.46 (14)	C23—C22—C21	120.30 (14)
C13—C8—C7	119.84 (14)	C27—C22—C21	120.33 (14)
C9—C8—C7	120.67 (14)	C22—C23—C24	120.52 (14)
C10—C9—C8	120.35 (16)	C22—C23—H23	119.7
C10—C9—H9	119.8	C24—C23—H23	119.7
C8—C9—H9	119.8	C25—C24—C23	119.76 (15)
C11—C10—C9	120.00 (16)	C25—C24—H24	120.1
C11—C10—H10	120.0	C23—C24—H24	120.1
C9—C10—H10	120.0	C24—C25—C26	119.79 (15)
C10—C11—C12	119.96 (15)	C24—C25—H25	120.1
C10—C11—H11	120.0	C26—C25—H25	120.1
C12—C11—H11	120.0	C27—C26—C25	120.56 (15)
C11—C12—C13	120.14 (16)	C27—C26—H26	119.7
C11—C12—H12	119.9	C25—C26—H26	119.7
C13—C12—H12	119.9	C26—C27—C22	120.02 (15)
C8—C13—C12	120.07 (15)	C26—C27—H27	120.0
C8—C13—H13	120.0	C22—C27—H27	120.0
C12—C13—H13	120.0	N2—C28—S2	178.13 (15)
			
C6—C1—C2—C3	−0.1 (2)	C20—C15—C16—C17	−0.1 (2)
C1—C2—C3—C4	1.0 (2)	C15—C16—C17—C18	0.4 (2)
C2—C3—C4—C5	−1.4 (2)	C16—C17—C18—C19	−0.3 (2)
C3—C4—C5—C6	0.8 (2)	C17—C18—C19—C20	−0.1 (2)
C2—C1—C6—C5	−0.4 (2)	C16—C15—C20—C19	−0.3 (2)
C2—C1—C6—C7	177.04 (14)	C16—C15—C20—C21	178.89 (13)
C4—C5—C6—C1	0.1 (2)	C18—C19—C20—C15	0.4 (2)
C4—C5—C6—C7	−177.57 (13)	C18—C19—C20—C21	−178.82 (12)
C14—N1—C7—C6	−94.4 (8)	C28—N2—C21—C20	−153.8 (7)
C14—N1—C7—C8	30.4 (8)	C28—N2—C21—C22	80.8 (7)
C1—C6—C7—N1	9.4 (2)	C15—C20—C21—N2	−13.98 (19)
C5—C6—C7—N1	−173.10 (13)	C19—C20—C21—N2	165.23 (12)
C1—C6—C7—C8	−114.46 (16)	C15—C20—C21—C22	109.54 (16)
C5—C6—C7—C8	63.05 (18)	C19—C20—C21—C22	−71.24 (17)
N1—C7—C8—C13	123.86 (15)	N2—C21—C22—C23	−123.47 (15)
C6—C7—C8—C13	−111.81 (15)	C20—C21—C22—C23	112.18 (15)
N1—C7—C8—C9	−58.43 (18)	N2—C21—C22—C27	57.70 (18)
C6—C7—C8—C9	65.90 (19)	C20—C21—C22—C27	−66.65 (17)
C13—C8—C9—C10	0.9 (2)	C27—C22—C23—C24	−1.0 (2)
C7—C8—C9—C10	−176.82 (14)	C21—C22—C23—C24	−179.89 (13)
C8—C9—C10—C11	−0.4 (2)	C22—C23—C24—C25	0.6 (2)
C9—C10—C11—C12	0.1 (2)	C23—C24—C25—C26	0.2 (2)
C10—C11—C12—C13	−0.3 (2)	C24—C25—C26—C27	−0.7 (2)
C9—C8—C13—C12	−1.1 (2)	C25—C26—C27—C22	0.2 (2)
C7—C8—C13—C12	176.66 (13)	C23—C22—C27—C26	0.6 (2)
C11—C12—C13—C8	0.8 (2)	C21—C22—C27—C26	179.47 (13)
C7—N1—C14—S1	−176 (100)	C21—N2—C28—S2	−168 (4)
